# Rapid Prototyping of a High Sensitivity Graphene Based Glucose Sensor Strip

**DOI:** 10.1371/journal.pone.0145036

**Published:** 2015-12-17

**Authors:** Farshad Tehrani, Lisa Reiner, Behzad Bavarian

**Affiliations:** 1 Keck’s Advanced Materials Laboratory, Manufacturing Systems Engineering & Management, California State University of Northridge, Northridge, California, United States of America; 2 Corrosion Research Laboratory, Manufacturing Systems Engineering & Management, California State University of Northridge, Northridge, California, United States of America; US Naval Reseach Laboratory, UNITED STATES

## Abstract

A rapid prototyping of an inexpensive, disposable graphene and copper nanocomposite sensor strip using polymeric flexible substrate for highly sensitive and selective nonenzymatic glucose detection has been developed and tested for direct oxidization of glucose. The CuNPs were electrochemically deposited on to the graphene sheets to improve electron transfer rates and to enhance electrocatalytic activity toward glucose. The graphene based electrode with CuNPs demonstrated a high degree of sensitivity (1101.3±56 μA/mM.cm^2^), excellent selectivity (without an interference with Ascorbic Acid, Uric Acid, Dopamine, and Acetaminophen), good stability with a linear response to glucose ranging from 0.1 mM to 0.6 mM concentration, and detection limits of 0.025 mM to 0.9 mM. Characterization of the electrodes was performed by scanning electron microscopy (FESEM and SEM). The electrochemical properties of the modified graphene electrodes were inspected by cyclic voltammetry (CV), electrochemical impedance spectroscopy (EIS), and amperometry.

## Introduction

Current glucose sensor technology relies mostly on enzymatic sensing strips that require repeated painful pricking methods to measure glucose concentration. The typical range of sensitivity for a blood glucose sensor is from 1.0 mM to 60 mM [1]. Non-diabetic and diabetic blood glucose levels fall within this range. A different approach for glucose determination is to use other fluid media. Saliva and tear fluid are alternatives to blood, but due to the low concentrations of analytes present in saliva, they require a very sensitive detection system. Tear glucose concentration is roughly 1/10 of the concentration of blood glucose—for saliva, the glucose concentration is even lower. Therefore, the commercial blood glucose sensors are not able to detect glucose in saliva or tears effectively. The primary focus of this research was to deliver a simple, reliable and accurate device that does not depend on chronic, invasive finger pricking, and does not rely on blood to determine glucose levels. An electrochemical sensor with a high degree of sensitivity and a desirable sensing range is achievable if it is made from graphene with a proper functionalization. Electrochemically deposited Copper nanoparticles were used for functionalization of the working electrode. A linear range suitable for detecting blood glucose levels in a human tear is obtained with significantly improving sensitivity below the 1.0 mM detection limit.

The performance of non-enzymatic glucose sensors relies mostly on two factors: the efficient electron transfer rate and an excellent catalytic material [2]. Graphene’s properties have been the focus of many recent studies and graphene is expected to significantly surpass the functionality of present materials in many applications. Graphene has been attributed with a large surface area, based on theoretical calculations, 2630 m^2^/g for a single layer [3], excellent thermal conductivity (k = 5 × 10^3^ W/mK) [4], a fast electron transfer rate, high electrical conductivity (σ = 64 mS/cm), and a large number of other material parameters such as mechanical stiffness, strength and elasticity. Some of these properties are derived from their delocalized π bonds above and below the basal plane. Similar to carbon nanotubes (CNTs), functionalized graphene due to great surface area (both sides are available) has shown remarkable capacities in detection of nanoscale biomolecules such as glucose as it can also be enhanced with the metal nanoparticles [5]. Furthermore, the performance of the graphene based biosensors is highly dependent on the fabrication method for producing the graphene. Multiple approaches have been used to produce graphene, such as chemical vapor deposition (CVD), molecular beam epitaxy, mechanical exfoliation, thermal exfoliation and chemical exfoliation [4]. Yet, with these approaches additional processing is required to effectively transfer the material to a useful substrate such as a flexible polymer substrate.

Improving the catalytic reactions for the electrode can be done by deposition of metal nanoparticles (NPs) on to the highly conductive surface of graphene sheets. This produces composites with larger active surface areas and improved electron transfer rates, making an ideal material for the fabrication of electrochemical sensors [6, 7]. Gold, silver, platinum, and nickel alloys have been used as catalytic materials for non-enzymatic glucose sensors [8–10] and they are capable of high sensitivity, however, poor selectivity and higher costs are limiting factors when compared to copper and its oxides, which have both high electrocatalytic activity and low cost [2]. Various copper nanostructures (nanoparticles, nanorods, nanocubes, nanodisks and nanowires) give large surface-to-volume ratios, fast electron transfer rate, superior catalytic ability and good sensing performance for non-enzymatic glucose sensors [11–20]. Previous research for copper nanostructured sensors [14–16, 21] has reported good performance. Further improvements have been made using the graphene/CuNPs material for the composite electrode in this work. These improvements can be attributed to the higher electron transfer rate due to optimal CuNPs population that can absorb and catalyze more reactive substance and increase the speed of oxidation. These enhanced properties will lead to even greater interest for industrial applications when mass-produced graphene with high quality and performance can match laboratory prototypes.

## Materials and Methods

Nano graphene platelets in powder form were obtained from Angstrom Materials LLC (N008-100-05). D-Glucose, Uric Acid, and Ascorbic Acid was purchased from sigma. All aqueous solutions were prepared using deionized water, using a Millipore water purification system with a minimum resistivity of 18.0 MΩ-cm. The electrode morphology and microstructure of the graphene and nanoparticles were characterized by scanning electron microscopy (SEM, JEOL JSM-6480LV, FESEM, Zeiss Ultra 55). Electrochemical measurements and resistance were performed using Gamry potentiostatic equipment, an electrochemical analyzer and multiple software packages (EIS300, PHE200, VFP600). The electrochemical measurements were based on a conventional three-electrode system with an Ag/AgCl as the reference electrode and PRG/CuNPs as the working electrode and bare graphene as the counter electrode. Electrochemical Impedance Spectroscopy (EIS) in 100 mM KCl solution containing 2mM K3[Fe(CN)6] + 2mM K4[Fe(CN)6] in the frequency range of 0.1 Hz to 100 kHz was used to obtain information on electron transfer rates between the electrolyte and the electrode surface. EIS normally includes a semicircular part and a linear part. The semicircular part at higher frequencies corresponds to the electron transfer limited process, and the diameter is equivalent to the electron transfer resistance (R_ct_), which normally reflects the charge transfer rate at the surface of the working electrode. The linear part at lower frequencies corresponds to the diffusion process. A straight line indicating Warburg resistance and the diffusion-limiting step in the electrochemical process [22] is a sign of good electrode conductivity. Cyclic Voltammetry (CV) was used for further investigation on the electrochemical behavior of the sensor strips. Ultimately, chronoamperometry experiments were used in order to study the amperometric response of the sensor toward different glucose concentrations.

### Fabrication of the electrodes

**Fig 1:**
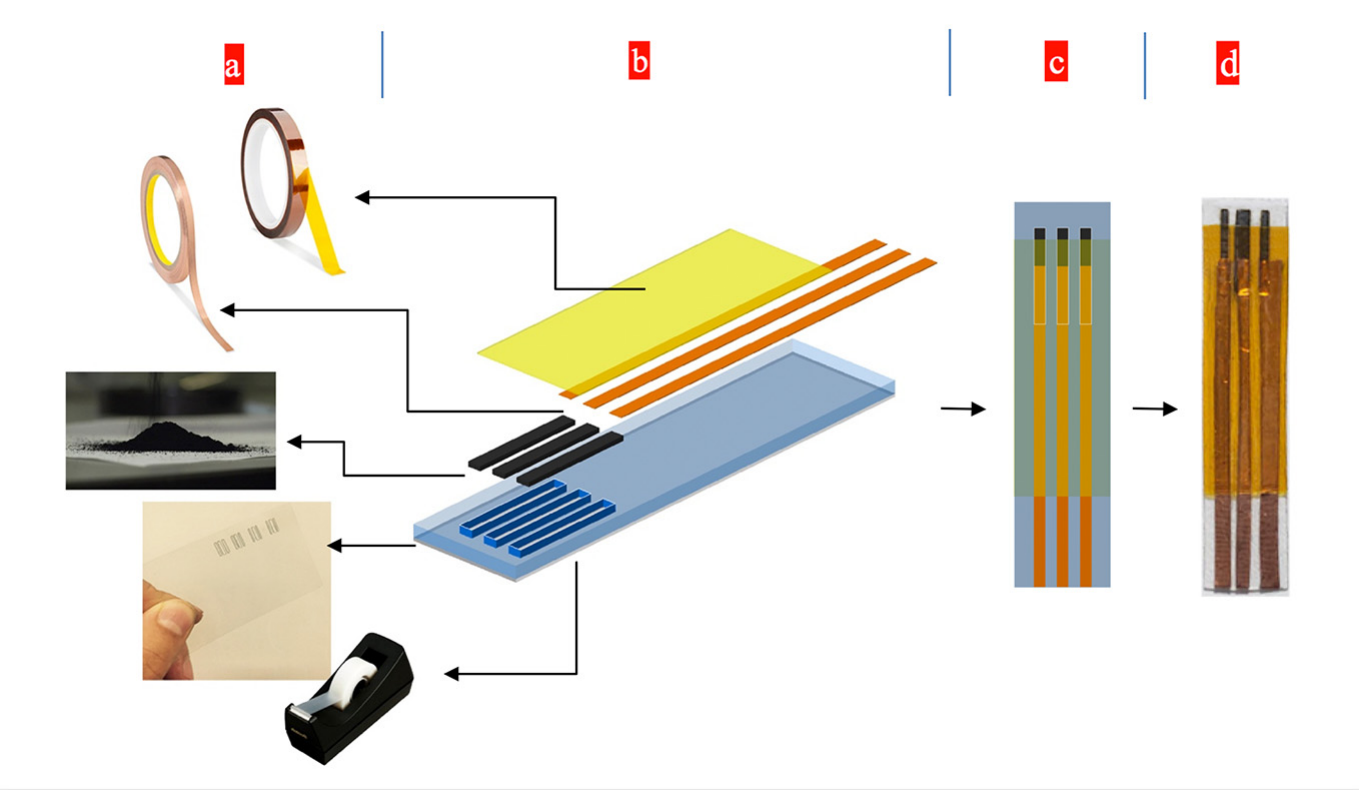
The rapid prototyping process of a disposable glucose sensor strip. a) raw materials from top to bottom, a thin Kapton tape used as the passivation layer; Copper tape used to form the sensor pad, Graphene powder used to form the three electrodes; PVC sheet with three laser-patented holes used to mask the substrate; Scotch tape used as the adhesive substrate, b) A drawing of the layers during assembly with the Scotch tape being masked. c) A drawing of the final sensor strip, ready for further modifications. d) A real sensor strip prototype, ready for further modifications.

For this investigation, simple processing integrates inexpensive materials (adhesive tape, graphene powder, copper tape, and silver conductive paste) to form the Graphene three electrode base. First, a PVC mask was prepared by removing a three-electrode channel into the PVC sheet using a laser engraving machine. Second, one side of the PVC sheet was taped using regular scotch tape, forming a flexible substrate with an adhesive three-electrode channel. Third, the graphene powder (≈0.25 mg for making a series of 4 strips) was placed on the three-electrode adhesive channel and physically rubbed against the surface of the adhesive Scotch Tape (SCT) in a gentle manner to form the uniform Physically Rubbed Graphene (PRG) electrodes. In the next two steps, electrodes were copper taped and then passivated by using a thin Kapton tape. Silver glue was used to enhance better connectivity in the copper-graphene intersection. An Ag/AgCl reference electrode was formed by gluing the tip of the electrode on the left side of the strip using a silver-silver chloride paste. The strip was stored in an ambient temperature for one day, for the paste to completely dry. An illustration of layers and the final strip is shown in the Fig 1. The exposed area of the working electrode (the electrode in the middle), was adjusted to be 0.19 cm^2^ (equivalent to the area of a circle with a diameter of 5mm). The processing method for the prototyping requires only several minutes of processing time at room temperatures, enables direct fabrication on flexible polymer substrates without an additional transfer step, and delivers strong surface adhesion between the few graphene layers and the flexible polymer substrates. Following the fabrication of the SCT/PRG base, the electrode was rinsed to remove loose graphene flakes and dried with low pressure argon gas to prepare for electrochemical deposition of copper nanoparticles.

### Modification of the Working electrode

CuNPs were electrochemically deposited on the surface of the working electrode. Since the sensitivity and the linear range of the sensor is highly dependent on the size and population density of CuNPs, the deposition of Copper nanoparticles on the working electrode is optimized systematically to achieve the smallest possible nanoparticles with a great population density. A large copper population will maximize the output signal of the biosensor due to an increased chemical reaction between the D-Glucose molecule and the CuNPs. The applied voltage during deposition improves the graphene surface and controls the electrodeposition parameters that contribute to optimal NP population with uniform distribution [23]. The formation of nuclei strongly depends on the interaction between the copper and the graphene. The abundant surface functional groups (−OH, C–O–C, and −COOH) on graphene provide reactive sites for the nucleation and binding of metals [10]. According to Grujicic, and Pesic [23], the three factors of applied potential, solution concentration of the Copper sulfate and Sodium sulfate along with the deposition time were controlled for an optimum result. In order to obtain an optimized size and population density of the CuNPs, four DC potentials of -0.2 v, -0.4 v, -0.6 v, and -0.8 v vs. Ag/AgCl were applied to the working electrode immersed into a 5 mM CuSO4 + 50 mM Na2SO4 solution for 350 seconds. The size and population of the CuNPs on Graphene sheets were observed using FESEM of the samples. Amperometry experiments of the strips was carried out to evaluate the performance of each strip according to its deposition process.

## Results and Discussion

### Characterization

**Fig 2:**
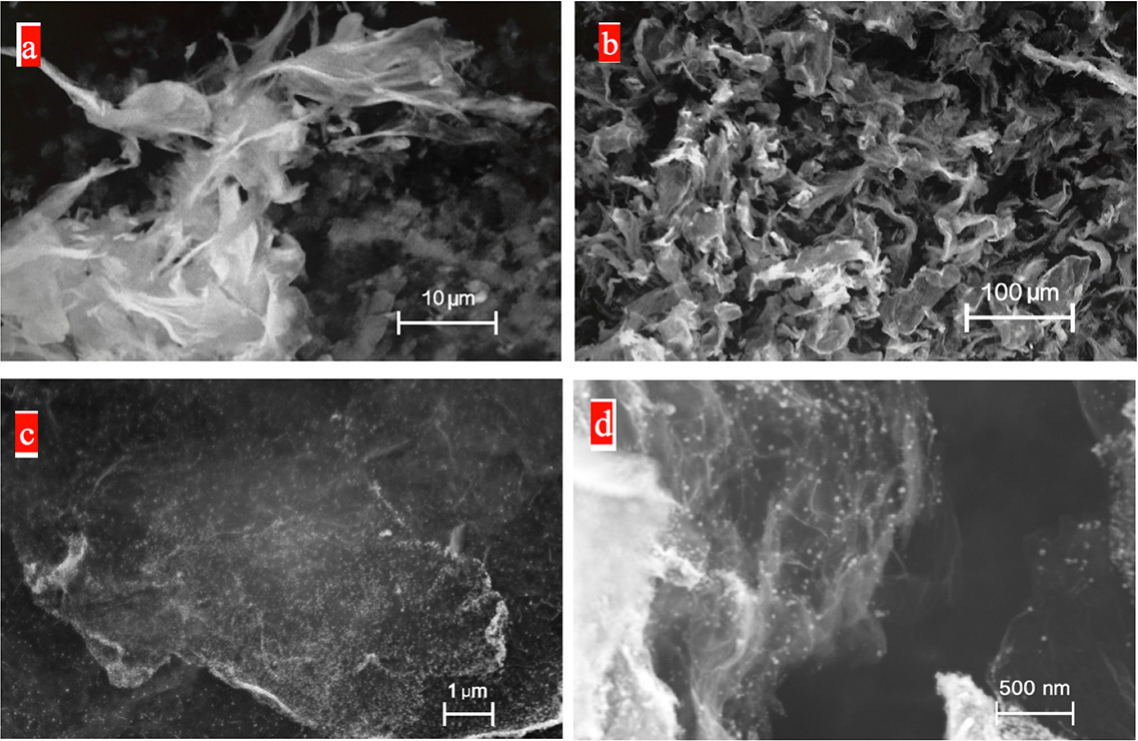
a & b) SEM of bare PRG sheets immobilized on a scotch tape. c & d) FESEM images of Copper nanoparticles electrochemically deposited on the PRG sheets.

Fig 2A & 2B show the SEM images of the irregular PRG that are vertically aligned on the adhesive surface of the SCT, creating a network of interconnected graphene sheets. Fig 2C & 2D, taken after the electrochemical deposition process of CuNPs on the PRG, show the FESEM images of the Copper nanoparticles are uniformly deposited on the graphene sheets. Among several applied potentials at the electrodeposition process for deposition of CuNPs on the SCT/PRG electrode, the applied potential of -0.60 v (vs. Ag/AgCl) demonstrated both the greatest amperometric signal response of the sensor, and formation of the finest CuNPs on the PRG sheets. Therefore, the optimal electrochemical deposition process results were identified to be achieved at a -0.60 v potential with a deposition time of 350 seconds using a 5 mM CuSO4 + 50 mM Na2SO4 solution without stirring of the solution.

**Fig 3:**
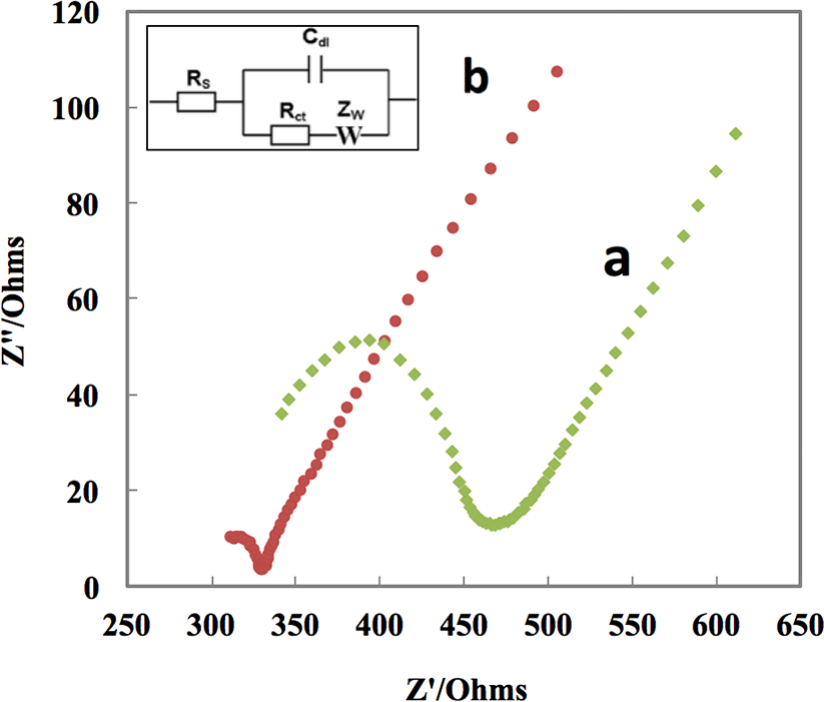
Nyquist plot of EIS for a) the bare SCT/PRG, and b) the SCT/PRG/CuNPs electrode; Inset is a Randles circuit.

In order to characterize the charge transfer behavior of the working electrode, the EIS tests were conducted. The Nyquist plots (Fig 3) of the bare SCT/PRG electrode and the SCT/PRG/CuNPs electrode were compared. According to the Randles equivalent circuit (Fig 3, inset), it can be seen that the R_ct_ for the modified electrode with CuNps (SCT/PRG/CuNPs) was smaller than the bare graphene electrode (SCT/PRG); demonstrating that the SCT/PRG/CuNPs electrode has a higher charge transfer rate than the bare graphene electrode. The copper nanoparticles are acting as electron mediators in the electron transfer process [19]. Accordingly, the bare SCT/PRG indicates a resistance of ≈151 Ω to the charge transfer at the surface of the working electrode and a R_s_ ≈288 Ω. After deposition of copper nanoparticles, the charge transfer of the electrode was reduced to 49 Ω, and roughly the same R_s_≈288 Ω was calculated.

**Fig 4:**
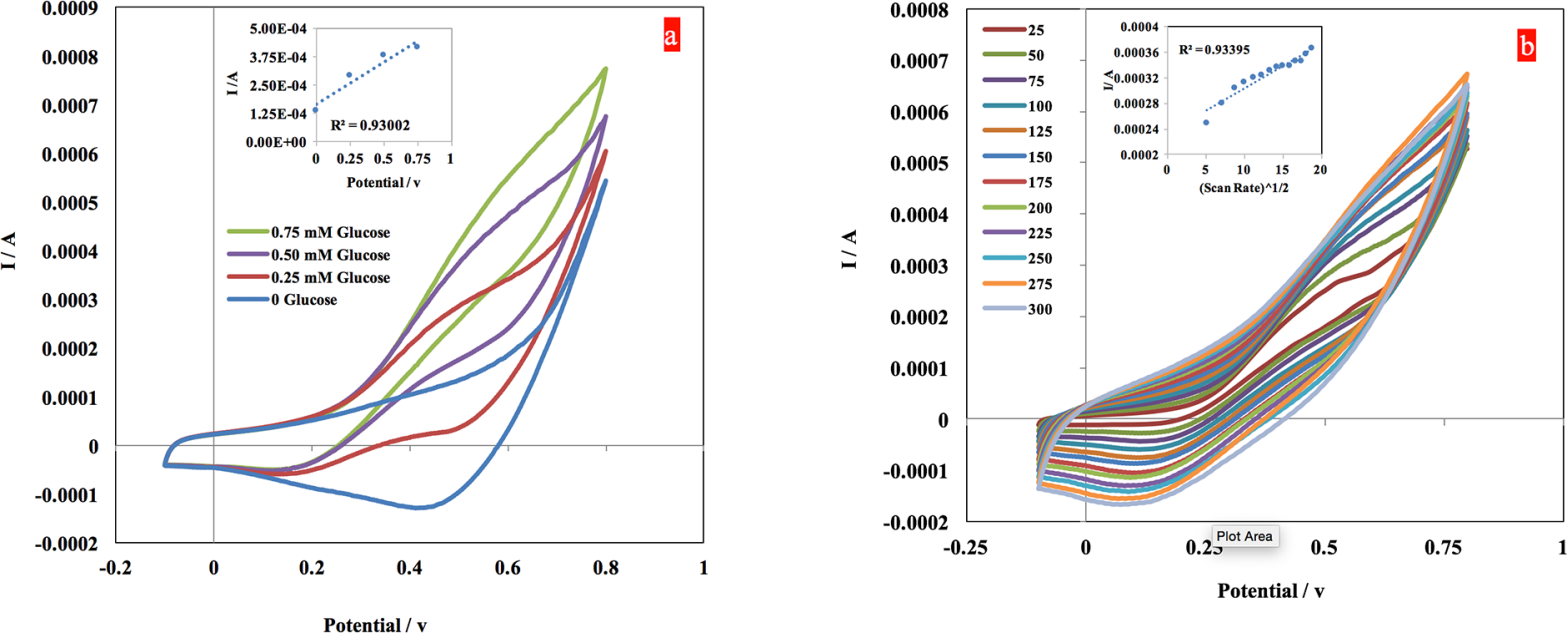
a) Cyclic voltammetry at a scanning rate of 100 mV/s in a 0.1 M NaOH solution that is used to characterize the electrochemical behavior of the PRG/CuNPs composite in the absence and presence of of 2 mM D-Glucose. b) Cyclic voltammetry performed to characterize the electrochemical behavior of the composite electrode in 0.1 M NaOH + 0.25 mM glucose at several scan rates ranging from 50 mV/ to 300 mV/s, with 25mV/s steps.

A comparison of the electrochemical behavior of the PRG/CuNPs composite was investigated by conducting a cyclic voltammetry (CV) experiment on the sensor in 0.1 M NaOH solution at a scan rate of 100 mV/s with successive addition of 0.25 mM, 0.50 mM, and 0.75mM of glucose. The oxidation of glucose starts at approximately 0.25 v, reaching an obvious shoulder peak at roughly 0.50 v, with the current continuing to increase with the increase in the potential (Fig 4A). The effect of scan rate on the oxidation of glucose molecules using the SCT/PRG/CuNPs electrodes was investigated by preforming multiple cyclic voltammetry experiments with different scan rates, starting from 25 to 300 mV/s, with a scan step of 25 mV/s (Fig 4B). A linear correlation (R^2^ = 0.93) between the anodic peak current and the square root of the scan rate was established (Fig 4B, inset plot), indicating the controllability of the glucose oxidation by adsorption of the molecules on the surface of the working electrode.

### Glucose sensing

**Fig 5:**
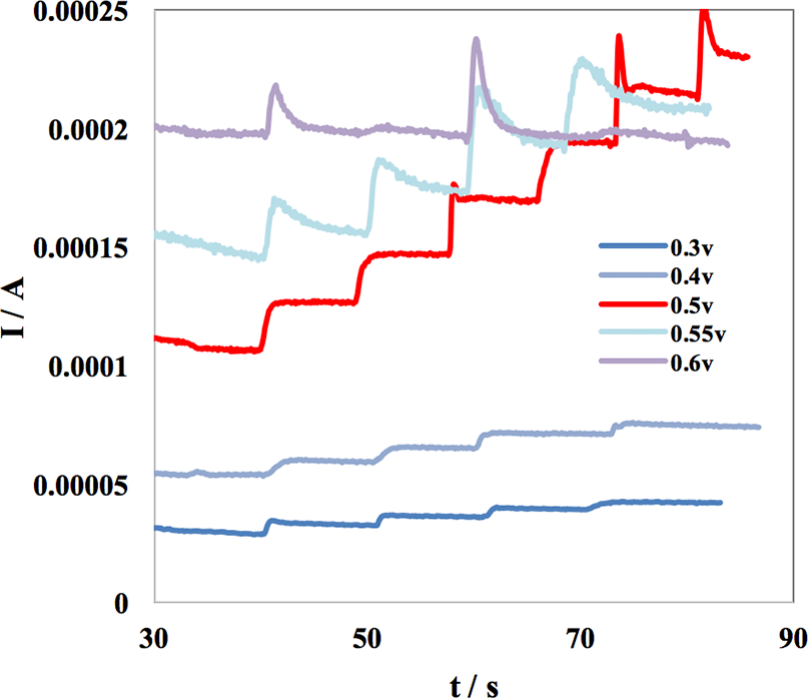
Amperometric response of sensor at different applied potential.

In order to obtain an optimized sensing potential, 5 different amperometric experiments were performed on one sensor using applied potentials of 0.30 v, 0.40 v, 0.50 v, 0.55 v, and 0.60 v, with successive additions of 0.1 mM glucose in a 100 mM NaOH solution. Results plotted in Fig 5 suggests 0.50 v to be the optimal applied potential because it produces the greatest step-like signal response upon the addition of glucose.

**Fig 6:**
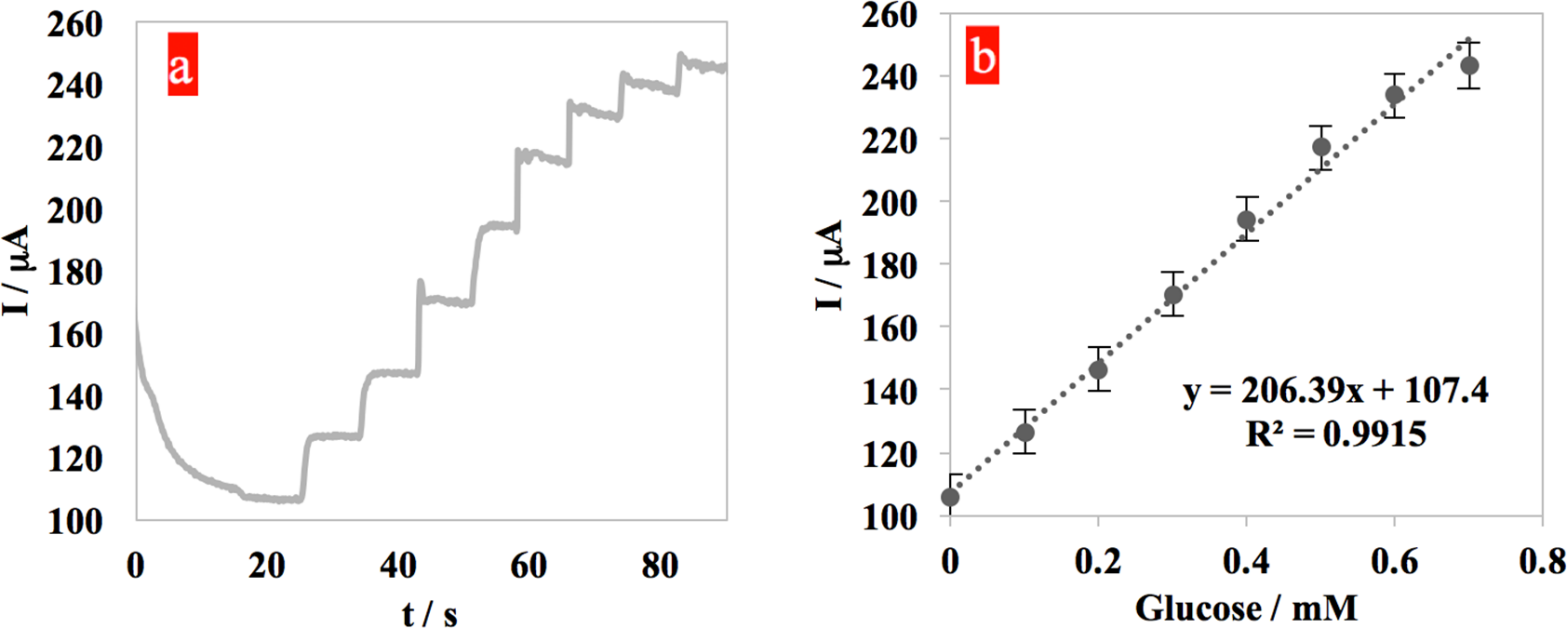
a) Amperometric response of the sensor with successive additions of 1mM D-glucose to the 0.1 M NaOH solution at the constant potential +0.50 v. b) Tear glucose range calibration curve.

In order to evaluate the amperometric behavior of the sensor, an apmerometry experiment on the sensor was performed at the sensing potential of 0.50 v in a 0.10 M NaOH solution. In 25 seconds (the time required to achieve a stable current) after the beginning of the test, D-Glucose (0.1 mM) was added every 8 seconds to the solution to observe the amperometric response of the system. Step-like current responses were apparent within 2 seconds (the response time) upon addition of 0.10 mM glucose in the detection range of 0.1 mM to 0.7 mM (Fig 6A). Therefore, a linear relationship between the current and the glucose concentration in that range was established (Fig 6B). This range is significant for glucose determination using tear fluid. The linear regression equation is given by y = 206.39x+107.4; R^2^ = 0.998. The glucose level at a current of 150 μA, for example, would be (y-b)/m or (150–107.4) μA/206.39 = 0.21 mM. The amperometry data revealed detection limits of 0.025 mM to 0.9 mM. The sensitivity was calculated to be 1101.3±56 μA/(mM.cm^2^). The rough mechanism for the oxidation of glucose in alkaline media at the copper modified electrode is believed to be the following: copper exposed to NaOH will oxidize into CuO [Cu + 2OH^−^ → CuO + H_2_O + 2e^−^]. Subsequently, CuO is electrochemically oxidized to Cu(III) species such as CuOOH or Cu(OH)_4_^−^ [CuO + OH^−^ →CuOOH or CuO + H_2_O + 2OH^−^ → Cu(OH)_4_^−^ + e^−^]. Finally, glucose is oxidized by the Cu(III) species and forms hydrolyzate gluconic acid [Cu(III) + glucose → gluconolactone + Cu(II)] [Gluconolactone → gluconic acid (hydrolysis)] [12, 14]. For comparison, glucose sensor performance has been reported in multiple published papers [5, 12, 14, 24–26]; several are listed in Table 1. The sensitivity of SCT/PRG/CuNPs using inexpensive, versatile, and flexible Scotch Tape, surpasses many of these electrode materials, which take advantage of highly conductive but expensive and inflexible glossy carbon substrate.

**Table 1:** Comparison of performance for several electrodes.

Electrode materials	Working potential vs. Ag/AgCl (V)	Sensitivity (μA/mM.cm^2^)	Reference
*CuO nanoplatelets*	0.55	3491	[14]
*CuO/graphene/GCE*	0.55	1360	[12]
*CuNWs/GTE*	0.60	1100	[2]
*CuNPs/PRG/SCT*	0.50	1101±56	This paper
*Cu flowers/GCE*	0.70	65.6	[15]
*Cu/CNT/GCE*	0.35	50.5	[16]

**Fig 7:**
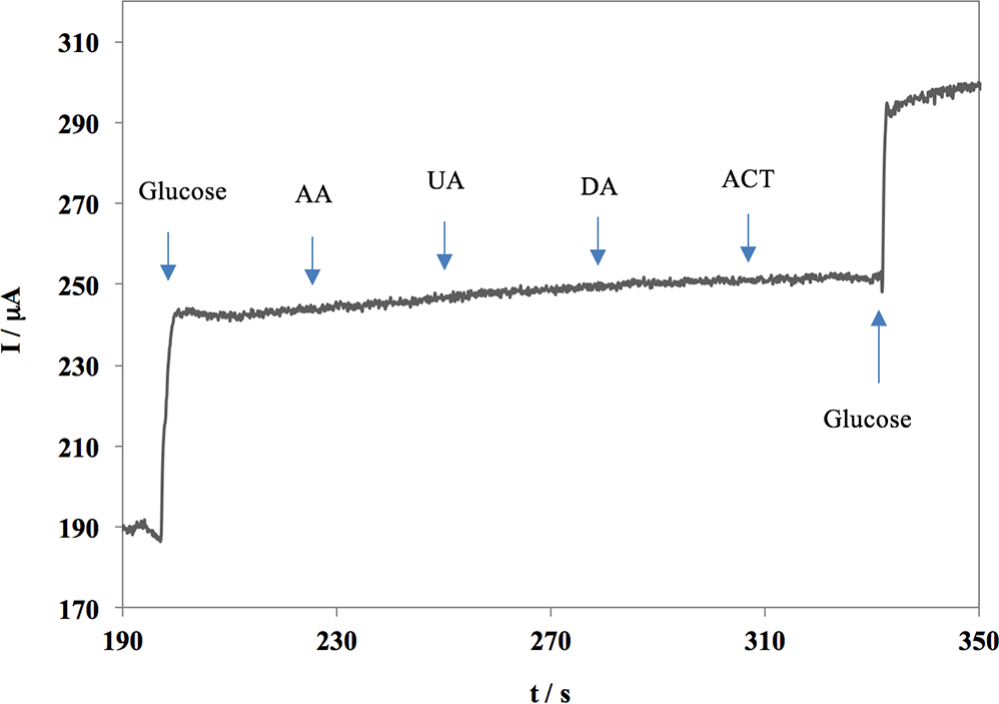
Amperometric response of the sensor against interferents found in the human serum as compare to 0.3 mM of glucose.

The effect of 250 μM Ascorbic Acid (AA), 250 μM Uric Acid (UA), 100 μM dopamine (DA), and 100 μM acetaminophen (ACT), as electrochemically active interferents found with glucose in human serum, was investigated on the sensor. This was carried out by systematically examining the amperometric response of the sensor upon successive addition of these interferents with their respective concentrations, as compared to 0.3 mM glucose in the order shown in Fig 7. The amperometric response of the sensor demonstrates no significant signals that can be observed for the interfering species. This is while obvious glucose oxidation signals were obtained before and after the addition of interferents. Therefore, the SCT/PRG/CuNps is highly selective towards glucose while the effects of AA, UA, DA, and ACT on the sensor were negligible.

**Fig 8:**
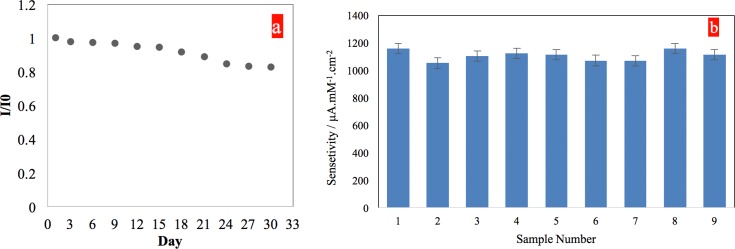
a) Stability of the sensor during a 30-day period with a total of 10 consecutive amperometric experiments. b) Reproducibility of the sensor among 9 samples indicating roughly 91% reproducibility with average sensitivity of 1106.9 μA/mM cm2 and a standard deviation of 37.7.

The reproducibility and stability of the sensor were investigated systematically. In order to investigate the reproducibility of the sensor, first, nine identical samples were fabricated and stored in normal and similar ambient condition. Next, chronoamperometry experiments were performed on the nine samples by measuring their output current signals while adding 0.1 mM of glucose into a 100 mM NaOH solution while stirring. From the amperometric responses a reproducibility of ≈91% with an average sensitivity of 1106.9 μA/mM cm^2^ and a standard deviation of 37.7 was calculated from the 9 sensor samples (Fig 8B), suggesting a relatively high reproducibility rate. The stability of the sensor was studied by performing 10 consecutive amperometric (1 experiment/ 3 days) experiments on one single sensor strip. I/I_0_ (I_0_ = initial current response on the first experiment) that was used as the stability measure of the sensor shows only a 17.2% of signal loss in the sensor strip (Fig 8A) after 10 times of consecutive use over a 30-day period. The small percentage of the signal loss in the sensor indicates a relatively high stability of the sensor that can be attributed to the stability of the SCT/PRG/CuNPs electrode, both in the ambient condition and in the basic solution.

## Limitation

The major limitation associated with our work is related to the fact that experiments were conducted in an in-vitro setup. However, this does not change the main findings in the study, since the focus of this work in establishing a robust linear amperometric correlation between glucose concentration and signal response was identified. Nevertheless, a rigorous in-vivo study of the biosensor is needed to evaluate its performance on actual human tear samples.

## Future Work

The concept of rapidly prototyping a highly sensitive graphene-based glucose sensor strip was proven in this study. This opens up an opportunity for a real application of the facile but competent biosensor strip, using a human tear sample. However, some challenges remain, including the direct sample collection by using the sensor strip without causing any harm to the delicate eye. These challenges should be addressed in a rigorously designed study of the strip as the future of this research. Such a study would involve non-invasive experiments for glucose sensing being performed on animal eyes, and subsequently, a clinical study of human eyes and tear samples.

It should be noted that the solid state application of graphene powder on Scotch Tape, or literally any other adhesive surface, is a versatile method of utilizing this wonder material for useful prototyping of practical devices. Additionally, the Graphene-Scotch Tape strip developed here should be seen as a versatile platform on which the application of variety of functionalization methods on it would be feasible. Therefore, rapid prototyping of conceptual graphene-based biosensor devices using such a platform is one way to advance this work. For instance, by using the same SCT/PRG platform, one may develop a SCT/PRG/Glucose Oxidase (GOx) bio-sensing strip by the GOx as the functionalizing agent. The use of the GOx instead the CuNPs is expected to allow a wider detection and linear range while delivering high level of sensitivity in the sensor

Furthermore, it should be considered that the compatibility of the sensor’s fabrication method with the screen printing technology would create opportunities for further development of the sensor in the following ways. First, the construction of a microfluidic channel on the strip would allow for a safe tear sample collection directly from the eye. Second, an accurate passivation and padding on the sensor strip would be expected to eliminate the human error during the fabrication step and increase the reproducibility rate of the sensor to percentages even higher than what is reported in this study. Third, mass production for commercialization of the biosensor.

## Conclusions

The behavior of most nonenzymatic glucose sensors is a direct function of the electrode material on which the glucose is oxidized. Recent efforts have exploited different materials and fabrication processes for these electrodes. Nanocomposite structure of graphene-metals or metal oxides offer large surface area for the oxidation of glucose. In this research, an inexpensive, disposable graphene and copper nanocomposite through a rapid prototyping process for highly selective nonenzymatic glucose detection was developed and tested for direct oxidization of glucose. The CuNPs effectively improve electron transfer rates and enhance electrocatalytic activity toward glucose. The graphene based electrode with CuNPs displayed good sensitivity, stability, linear range, detection limit, and selectivity against interfering molecules.

## Supporting Information

S1 File Raw data used in the study. (XLSX)
